# The Fatty Acid Transporter CD36 Mediates Uptake, Biodistribution, and Cardioprotection by Small Extracellular Vesicles From HEK293 Cells

**DOI:** 10.1002/jev2.70254

**Published:** 2026-03-17

**Authors:** Elias Sulaiman, Derek M. Yellon, Sean M. Davidson

**Affiliations:** ^1^ The Hatter Cardiovascular Institute University College London London UK

**Keywords:** biodistribution, cardioprotection, CD36, extracellular vesicles, ischaemia, mice, nanoluciferase, reperfusion

## Abstract

Extracellular vesicles (EVs) are nanoscale carriers of bioactive molecules that mediate intercellular communication. Small EVs (sEVs) have shown cardioprotective effects in models of myocardial infarction (MI), but their uptake and biodistribution remain incompletely understood. Here, we investigated NanoLuc‐labelled HEK293‐derived sEVs, assessing their uptake in cardiac cells, in vivo biodistribution, and effect on infarct size following cardiac ischaemia and reperfusion (IR) injury. We hypothesised that the scavenger receptor CD36, a lipid‐binding receptor expressed in both cardiomyocytes and endothelial cells, may mediate sEV uptake. sEVs were purified by size‐exclusion chromatography and tangential flow filtration. In vitro, cardiac endothelial cells internalised sEVs more efficiently than cardiomyocytes. In healthy mice, sEVs accumulated mainly in the lungs, liver, and spleen. However, when administered post‐IR, sEVs significantly reduced infarct size (from 58% ± 8% to 36% ± 3%, *p* < 0.05, *n* = 5). Inhibition of CD36 with sulfosuccinimidyl oleate impaired sEV uptake and abolished cardioprotection. These findings suggest HEK293‐sEVs may have therapeutic potential in MI and identify CD36 as a key mediator of their uptake and function.

## 1 Introduction

Extracellular vesicles (EVs) are nanosized vesicles enclosed by a lipid bilayer and containing a diverse cargo that facilitates intercellular communication (Davidson et al. 2023, Davidson and Yellon 2018, Pearce et al. 2025). Different subtypes of EV, such as exosomes and microvesicles, have been shown to be carriers of proteins and nucleic acids (mostly miRNAs), while the lipid membrane is comprised of cholesterol, phosphatidylserine and phosphatidylcholine (Ghadami and Dellinger 2023, Skotland et al. 2020). The MISEVs2023 (Minimal information for studies of extracellular vesicles) guidelines recommend that EVs are categorised either by size, origin, or composition. In general, EVs that have a diameter smaller than 200 nm are characterised as small extracellular vesicles, or sEVs (Welsh et al. 2024).

sEVs are primarily internalised by cells via endocytosis (either clathrin‐, caveolin‐, or lipid raft‐mediated), macropinocytosis, phagocytosis, or membrane fusion (Mulcahy et al. 2014). More than one uptake pathway can exist in the same cell, and these can differ between cell types (Mulcahy et al. 2014). After *in vivo* administration in rodents, sEVs exhibit a surprisingly short half‐life of only ∼5 min, and then are found primarily distributed to the lungs, liver, and the spleen (Kang et al. 2021, Driedonks et al. 2022). However, tracking of biodistribution remains challenging, and varies on the cell type of origin (Driedonks et al. 2022). Furthermore, it is unclear which uptake pathways are involved *in vivo*. An understanding of the molecular mechanisms that govern the *in vitro* and *in vivo* behaviour of sEVs is essential to aid in their development, and maximise their potential as therapeutic agents.

Several reports have shown that EVs—primarily sEVs—can provide therapeutic benefits in animal models of acute myocardial infarction (MI), in which animals are subject to transient ischaemia and reperfusion, followed by assessment of myocardial infarct size (Davidson et al. 2023, Pearce et al. 2025, Davidson et al. 2019, Davidson et al. 2017). For these experiments, sEVs have primarily been obtained from stromal cell sources (e.g., MSC (mesenchymal stem cells), iPSC (induced pluripotent stem cells), CPC (cardiac progenitor cells), and CDC (cardiosphere‐derived cells)). These EVs have been shown to successfully reduce the infarct size in animal models of myocardial ischaemia‐reperfusion (I/R) injury (Davidson and Yellon 2018, Davidson et al. 2017, Ciullo et al. 2022, Roefs et al. 2023). The mechanism is believed to involve kinase signalling pathways such as the PI3K/Akt or the mTOR pathway. However, there remains an important knowledge gap in the EV field to understand how different cell sources, with variable biological functions, can produce sEVs with cardioprotective potential.

A common component of the exosomal membrane phosphatidylserine (PS), a lipid known to play a role in cellular recognition via CD36 (Matsumoto et al. 2017). The lipid bilayer of sEVs contains various lipids—including cholesterol, phosphatidylcholine, sphingomyelin, and notably phosphatidylserine—which contribute to their biological behaviour. Several studies indicate that PS is important for the uptake of sEVs by recipient cells (Matsumoto et al. 2017). This underscores the functional significance of PS in vesicle‐cell interactions. Furthermore, liposomes enriched in PS, despite being considered biologically inert, have been shown to improve cardiac function and reduce scar formation in a non‐reperfused rat model of myocardial infarction (Harel‐Adar et al. 2011). Taken together, these findings suggest that PS on exosomal membranes may facilitate their uptake into cardiac cells or macrophages, potentially contributing to cardioprotective effects.

HEK293 cells are an immortalised cell line that are widely used in the EVs field to study vesicular biogenesis, trafficking, and for the development of methods to genetically modify EVs cargo. This is largely for practical reasons based on their rapid growth, ease of transfection, efficient transgene expression, and ability to grow in serum‐free medium, which has also led to them as the platform of choice to produce recombinant proteins and viral vectors (Tan et al. 2021). The cells do not appear to have pro‐angiogenic, immunomodulatory, or regenerative properties (Vo et al. 2024). Nevertheless, since they may still contain bioactive components such as lipids or miRNA, we decided to investigate whether sEVs from HEK293 cells have the potential to be cardioprotective in acute MI.

We also investigated whether CD36 is involved in the uptake of HEK293‐EVs into cells. The scavenger receptor CD36 is ubiquitously expressed in endothelial cells, macrophages, and cardiomyocytes, and is known to mediate the internalisation of oxLDL (oxidised LDL) particles, and function in uptake of long‐chain fatty acids such as phosphatidylserine and phosphatidylcholine (Jay and Hamilton 2018). Furthermore, platelet CD36 mediates interactions with endothelial cell–derived microparticles (Ghosh et al. 2008), and CD36 is important for binding of RBC‐EVs to monocytes (Danesh et al. 2018). There are notable structural and compositional similarities between sEVs and oxidised low‐density lipoproteins (oxLDL), including in the lipid components and surface motifs that may be recognised by the same receptors. Given the structural similarities between sEVs and oxLDL, and the fact that both phosphatidylserine and phosphatidylcholine are constituents of the sEVs lipid membrane, we hypothesised that CD36 plays an important role in the biological function of HEK293‐derived sEVs. We investigated this using sulfo‐N‐succinimidyl oleate (SSO), which irreversibly inhibits CD36.

Our findings revealed that HEK293‐sEVs are rapidly internalised by endothelial cells versus cardiomyocytes, are primarily localised in the lungs and liver of mice upon intravenous administration, and reduce the infarct size in a mouse model of acute M I/R. Importantly, we showed that inhibition of CD36 in vitro reduced sEVs uptake in both cell types, led to a diminished internalisation in the lungs, and abrogated their cardioprotective properties.

## 2 Materials and Methods

### 2.1 Animals

Male C57BL/6 mice (12–14 weeks old) and male Sprague‐Dawley rats (250–350 g) were obtained from Charles River, United Kingdom. Animals were housed in a controlled temperature environment with a 12‐h light/dark cycle. Food and water provided *ad libitum*. All experiments were conducted under the United Kingdom Animals (Scientific Procedures) Act 1986 Amendment Regulations 2012. All procedures were in accordance with the project licence PPL9987686, approved by the Home Office and the Animal Welfare and Ethical Review Board at UCL.

### 2.2 Cell Culture

HEK293 cells (Human embryonic kidney 293, acquired from ATCC) were cultured in Dulbecco's Modified Eagle Medium (DMEM, Gibco, 41966‐029) with 10% foetal bovine serum (FBS, Sigma‐Aldrich, F9665) and 1% penicillin/streptomycin (Sigma‐Aldrich, P4333). HUVEC (Human Umbilical Vein Endothelial Cells) were cultured in endothelial cell basal medium 2 (PromoCell, C‐22011) supplemented with the supplement mix (PromoCell, C‐39216). HCMEC (human coronary microvascular endothelial cells) and HCAEC (human coronary artery endothelial cells) were cultured in endothelial cell basal medium MV‐2 (PromoCell, C‐22022) supplemented with the supplement mix (PromoCell, C‐39226). All cells were incubated at 37°C and 5% CO_2_. All cells were passaged once they reached ∼80% confluency. HEK293 cells were detached with Trypsin (Gibco, 15090046) and all endothelial cells with Accutase solution (PromoCell, C‐41310).

### 2.3 Primary Adult rat Cardiomyocyte Isolation

Rat cardiomyocytes were isolated from adult rat hearts as previously described (Shah et al. 2022). On the day of isolation, rats were anaesthetised by intraperitoneal injection of 1 mL/kg pentobarbitone (Dolethal) with heparin 500UI. Then, hearts were excised and immediately perfused via the aorta with a perfusion buffer (10 mM glucose, 20 mM taurine and 10 mM creatine, 5.4 mM KCl, 1.4 mM MgCl_2,_ 130 mM NaCl, 0.4 mM Na_2_HPO_4_, 4.2 mM HEPES, maintained at 37°C and pH 7.4). Firstly, the heart was cleared of blood by perfusing with the perfusion buffer containing additional 750 µM CaCl_2_, followed by arresting the heart with the addition of 100 µµ EGTA in the buffer (without CaCl_2_), and lastly by digesting the tissue with the buffer including 100 µM CaCl_2_, 2 mg of protease and collagenase (2500 units/ 100 g of rat body weight). Rat cardiomyocytes were collected from the left ventricle and cultured *in vitro* with M119 medium supplemented with 1% penicillin/streptomycin, 2 mM carnitine, 5 mM creatinine, 5 mM taurine, and l‐glutamine. Cardiomyocytes were cultured in either a 6‐well plate or a white µClear bottom 96‐well plate (Greiner bio‐one, 655098), which were pre‐coated with 1.5% laminin (Invitrogen, 23017‐015).

### 2.4 Plasmid DNA Production and HEK293 Transfection

The pDB30 plasmid encodes CD63‐Nluc, where Nluc is a Nanoluciferase sequence that is added to the C‐terminus of CD63. This was generously provided by the Fussenegger lab (Kojima et al. 2018). JM101 bacteria (*E.coli* from Promega, L2005) were grown in lysogeny broth (LB) medium (5 g yeast extract, 10 g NaCl, 10 g tryptone, distilled water, pH adjusted to 7.0), and were transformed with pDB30 by heat shock as previously described (Sulaiman et al. 2025). Briefly, bacteria were incubated with the plasmid DNA pDB30 for 30 min on ice, followed by a 45‐s heat shock at 42°C. Then, they were again incubated on ice for 2 min before allowing their recovery for 60 min under shaking at 300 rpm at 37°C. Following their recovery, bacteria were grown on agar plates with ampicillin. pDB30 was then extracted by using the Plasmid Maxi prep kit (Qiagen, USA), and frozen until further use.

For the transfection of HEK293 cells, pDB30 was mixed with polyethylenimine (PEI, Sigma‐Aldrich) at a ratio of 1:4 and added to cells that were at ∼70%–80% confluency. Transfection was allowed overnight, after which cells were washed with PBS and cultured with fresh serum‐free DMEM for a further 24 h to allow for their production and release of sEVs.

### 2.5 Concentration of Small Extracellular Vesicles (sEVs)

Conditioned DMEM (without serum) was collected from HEK293 cells and sEVs were concentrated by tangential flow filtration (TFF) coupled with size exclusion chromatography (SEC). Briefly, media was centrifuged 10 min at 3,000 g, 4°C to remove cells and cell debris. Then, the supernatant was filtered with TFF (Vivaflow 50R by Sartorius, VF05H4) with a filtration membrane of 100,000 MWCO. The media was collected at a final volume of 20 mL and was further filtered with Vivaspin 20R and Vivaspin 2 tubes (100,000 MWCO filter, Sartorius) in the centrifuge. The concentrated media was collected and diluted with PBS to a final volume of 500 µL and was fractionated with the SEC column qEVs 35 nm (Izon Science, UK). All SEC fractions were stored at –80°C until further use.

### 2.6 Nanoparticle Tracking Analysis (NTA)

The size and concentration of HEK293‐sEVs were defined by nanoparticle tracking analysis (NTA). NanoSight (LM10‐HS, Malvern, UK) was utilised with a 488 nm violet laser module, where the camera level was set to 15 and the detection threshold to 5. Three recordings of 90 s were acquired for each sample and data were extracted with NTA 3.1 Build 3.1.54 and analysed.

### 2.7 BCA (bicinchoninic) Protein Assay

To assess the purity of HEK293‐sEVs isolations, a BCA protein assay for low concentrations (Abcam, ab207002) was conducted, according to the manufacturer's instructions. Briefly, 2 µL of the most particle‐rich SEC sample of HEK293‐sEVs was diluted in 148 µL of PBS, 150 µL of the BCA reagent working solution was added, the plate was shaken for 30 s at 500 rpm and incubated for 2 h at 37°C. Absorbance was measured at 562 nm using a FLUOstar plate reader (BMG Labtech, UK).

### 2.8 Dissociation‐Enhanced Lanthanide Fluorescence Immunoassay (DELFIA)

DELFIA (a modified ELISA assay), was utilised to detect the sEVs‐specific markers CD9, CD63, and CD81, as previously described (Takov et al. 2017, Takov et al. 2020). Samples were loaded on high‐binding 96‐well plates (R&D Systems, UK, DY990) and incubated overnight at 4°C. Then, wells were washed with DELFIA Wash buffer (PerkinElmer, 1244‐114) and incubated with 1% BSA/PBS for 1 h at room temperature (RT). Subsequently, the primary antibodies (BD Biosciences, USA) for CD9 (555370), CD63 (556019), and CD81 (555675) were loaded at a concentration of 1 µg/ml and incubation was allowed at RT for 2 h, after which wells were washed with DELFIA wash buffer and 0.25 µg/ml of the secondary biotinylated antibody (Abcam, ab98691) was added for 1 h at RT. Lastly, the streptavidin‐europium conjugate (PerkinElmer, 1244‐106) was diluted 1:1000 in DELFIA assay buffer (PerkinElmer, 1244‐30) and added to the wells for 1 h at RT, then wells were washed six times with the wash buffer before loading the DELFIA enhancement solution (PerkinElmer) and quantifying the fluorescence (arbitrary units, a.u.) with a PHERAstar plate reader (BMG Labtech). Settings for the plate reader were set at 337 nm excitation, 620 nm detection, 200 µs integration time and 60 µs lag time.

### 2.9 Luciferase Assay

HEK293‐sEVs were detected *in vitro* by using the Nano‐Glo Luciferase Assay System (N1120, Promega) as previously described (Sulaiman et al. 2025). 10 µL of SEC fractions or 1 µL of HEK293 cell lysate were added to a white 96‐well plate and incubated with 100 µL of the substrate in assay buffer (diluted at 1:50). Then, the plates underwent shaking for 3 min at 500 rpm and luminescence (measured as Nluc activity in arbitrary units) was measured with a FLUOstar plate reader (BMG Labtech, UK).

### 2.10 Cellular Uptake of HEK293‐sEVs

HCAEC, HCMEC, HUVEC, or adult rat cardiomyocytes were cultured in white, µClear, 96‐well plates (Greiner bio‐one, 655098) as previously described. To investigate the uptake of HEK293‐sEVs in these cell types of the cardiac tissue, 10^7^, 10^8^, and 10^9^ sEVs were incubated for 1, 4, and 24 h. After the incubation period, cells were washed three times with PBS, the Nluc activity was measured as described above.

To investigate the possibility that sEVs stick to the cell membrane rather than enter the cytosol, before quantifying Nluc, cells were treated with citric buffer for 5 min at RT (10 mM KCl, 135 mM NaCl, 40 mM citric acid, pH 3.0) (Li et al. 2020).

To study the effects of the CD36 fatty acid transporter in sEVs cellular uptake, HCMEC and rat cardiomyocytes were treated with the irreversible inhibitor SSO (sulfosuccinimidyl oleate sodium, Abcam, 135661‐44‐8) at 200 µM for 1 h before allowing the uptake of 10^9^ HEK293‐sEVs for 1 h. As a positive control, cells were pre‐treated with 25 µM chlorpromazine (CPZ, Sigma‐Aldrich, C8138‐5G) for 1 h, which inhibits clathrin‐mediated endocytosis, followed by the addition of 10^9^ HEK293‐sEVs for another 1 h (Ko et al. 2019).

### 2.11 Immunostaining for HEK293‐sEVs Cellular Uptake

HCMEC and adult rat cardiomyocytes were cultured as previously described and treated with fluorescently labelled HEK293‐sEVs to visualise uptake. CellMask Orange Fluorescent dye (ThermoFisher, C10045) was used to stain HEK293‐sEVs (Takov et al. 2017). Briefly, 7.5 µg/ml of the dye was added to 50 µL of HEK293‐sEVs to a final volume of 500 µL. Staining was allowed for 10 min at 37°C, after which the mixture was centrifugated at 14,000 g for 5 min (4°C) to remove the unbound, excess dye with Amicon Ultra Centrifugal tubes (100 kDa, Merck, UFC510008). Samples were washed three times with PBS to ensure sufficient removal of the free fluorescent molecules and the pellet of sEVs was added to the cells for 1 h at 37°C/5% CO_2_. Cells were cultured on coverslips (25 mm) in 6‐well plates. As a control group, to rule out the possibility that the molecules of this dye form micelles that co‐isolate with HEK293‐sEVs, the dye was diluted to the same concentration and final volume, and the pellet was also added to cells (Davidson et al. 2023).

Following the incubation time, cells were washed three times with PBS, and fixed for 5 min at 37°C with 4% paraformaldehyde in PBS containing 1:2000 Hoechst staining (ThermoFisher, 33342) to stain the nuclei. Samples were then imaged with a Leica TCS SP5 confocal microscope and images were analysed with ImageJ.

### 2.12 In Vivo Myocardial Ischaemia‐Reperfusion Injury (M I/R)

Mice were anaesthetised with intraperitoneal injection of 100 mg/kg pentobarbital and intubated to a rodent ventilator (MinVent, Type 845, Hugo Sachs Elektronik). Temperature was monitored throughout the experiment with a rectal thermometer and stabilised at 37°C. Mice were secured at a decubitus position, the chest was shaved, and the heart was exposed after opening the chest between the 3^rd^ and 4^th^ ribs at the 3^rd^ intercostal space. The left anterior descending artery (LAD) was identified and ligated with an 8–0 silk suture (Ethicon, W2775) for 40 min to induce ischaemia. Then, the snare was released to allow the reperfusion of the myocardium for 120 min. HEK293‐sEVs (10^10^ particles in 100 µL saline) or vehicle control (PBS in 100 µL saline) were injected intravenously (jugular vein) at the onset of reperfusion. After 2 h reperfusion, the hearts were stained with 4% Evan's blue and 1% TTC (triphenyl tetrazolium), cut in 5–6 slices, and incubated overnight in 10% formalin. These experiments were in agreement with the practical guidelines for preclinical studies on cardioprotection (Bøtker et al. 2018). Heart slices were scanned using a CanoScan LiDE 220 (Cannon), and the infarct size and area at risk were calculated with ImageJ.

To investigate the effects of the CD36 transporter in the cardioprotective effects of HEK293‐sEVs, 100 µL of 40 mg/kg of the irreversible SSO inhibitor (Harmon and Abumrad 1993), or vehicle control (DMSO), were injected intraperitoneally 1 h before the administration of the sEVs (i.e., 20 min before the introduction of ischaemia).

### 2.13 Biodistribution of HEK293‐sEVs in Mice

To understand the in vivo pharmacokinetics of HEK293‐sEVs, mice were injected intravenously with 10^10^ particles and biodistribution was allowed for 1 h. To investigate what proportion of HEK293‐sEVs are retained from the blood components in the capillaries of the organs and what proportion is truly internalised in the organs, a perfusion protocol was utilised as previously reported (Gage et al.; Wu et al.). Briefly, after sEVs biodistribution, a small cut was applied to the left atrium of the heart and a syringe with 50 mL of saline was inserted in the apex of the left ventricle to conduct whole‐body perfusion. To investigate if the CD36 transporter influences the biodistribution of HEK293‐sEVs, the SSO inhibitor (or DMSO) was administered at 40 mg/kg intraperitoneally 1 h before the sEVs. Throughout the experiment, mice were under general anaesthesia and intubated to an animal ventilator as previously described. At the end of the experiment, fluids (blood and urine) and organs (heart, lungs, liver, kidney, spleen, brain) were collected. Blood was added to a tube with heparin (1:100) to prevent clotting, and was centrifugated for 10 min at 10,000 rpm, 4°C to separate the plasma from the blood cells. All fluid samples were stored in ice until analysis and frozen at –80°C. Organs were snap frozen in liquid N_2_ and immediately frozen at –80°C until further analysis.

Before analysis, blood cells and organs were firstly lysed with a lysis buffer (1.75 g NaCl, 1% IGEPAL (Merck, I8896), 1.21 g Trizma base, 100 mL H2O final volume). Briefly, 100 µL of lysis buffer was added to the blood cells, 1 mL to the heart, lungs, liver, spleen, brain, and 2 mL to the liver and the kidneys. Organs were lysed on ice with a tissue homogeniser, and the liquid was centrifugated for 10 min at 10,000 rpm at 4°C. 10 µL of each sample (fluids and organs) were analysed with the luciferase assay as previously described to detect HEK293‐sEVs.

### 2.14 Statistical Analysis

Data plotted with GraphPad Prism 9 (GraphPad Software, San Diego, USA) and presented as mean ± SEM. One‐way ANOVA with Tukey's post‐hoc test was utilised to statistically analyse the data with threshold significance set at *P* < 0.05.

## 3 Results

### 3.1 Isolation and Characterisation of HEK293‐sEVs

HEK293 cells were transfected with the pDB30 plasmid DNA with the purpose of producing sEVs that express Nanoluciferase fused to the C‐terminus of CD63. This allows the HEK293‐sEVs that are produced to be detected with high sensitivity in vitro with a luciferase assay. We have previously shown that transfection with pDB30 leads to a successful cellular expression of the CD63‐Nluc construct and subsequent incorporation to the vesicular membrane. This transfection does not change the size and the size distribution of the modified vs the unmodified sEVs from HEK293 cells (Sulaiman et al. 2025). sEVs were concentrated from the HEK293 cell media via ultrafiltration (TFF coupled with SEC) and further characterised physically and biochemically.

**FIGURE 1:**
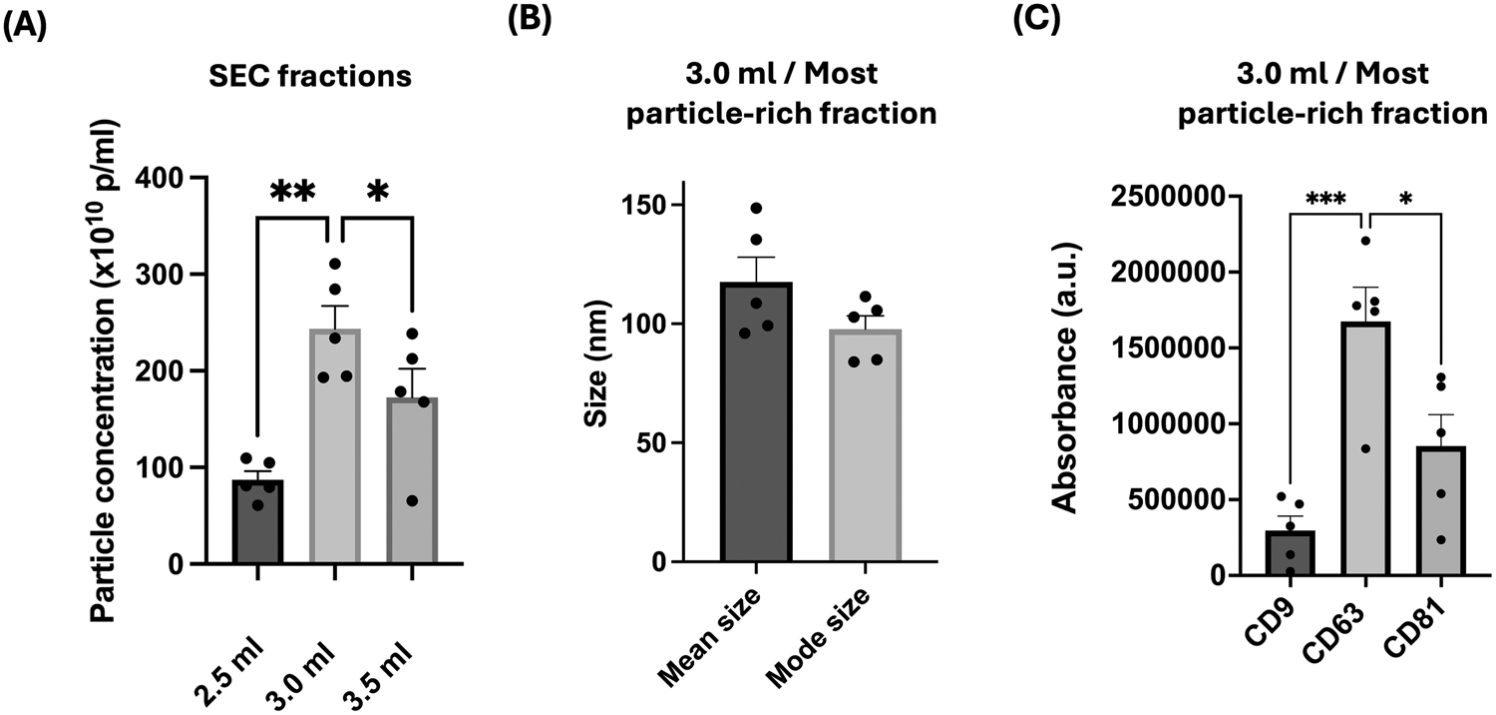
Physical and biochemical characterisation of HEK2930‐sEVs. Nanoparticle tracking analysis (NTA) shows that (A) the most particle‐rich fraction is eluted after 3.0 mL with size exclusion chromatography (SEC) with a concentration of (242 ± 21) ×10^10^ particles/ml with (B) a mean size of 118 ± 52 nm and mode size 98 nm ± 44 nm. (C) Fraction 3.0 mL has high levels of the three EVs‐specific markers, with a higher presence for CD63, as shown by DELFIA (Dissociation‐enhanced lanthanide fluorescence immunoassay). Data shown as mean ± SEM (*n* = 5). *p < 0.05, **p < 0.01, ***p < 0.001. HEK293: human embryonic kidney cells 293; sEVs: small extracellular vesicles.

The most particle‐rich fraction after SEC was eluted after 3.0 mL (or fraction 7) and had a concentration of (2.4 ± 0.2) × 10^12^ particles/ml (*n* = 4, Figure 1). The size distribution of the vesicles was narrow, with a peak at ∼108 nm (Figure S1A), and the mode size was at 97 nm ± 43 nm (*n* = 4, Figure 1). All three of the commonly used EVs markers CD9, CD63 and CD81 were present in the most particle‐rich fraction, with staining for CD63 being the greatest (*n* = 4, Figure 1). The most particle‐rich fraction was also the most luminescent one, which indicates that the concentration protocol utilised here results in one fraction that contains the majority of the smallest, luminescent vesicles or sEVs (Figure S1B). Lastly, according to the Webber calculations of particle/protein ratio as a measure of EV purity (Webber and Clayton 2013), this fraction was of ‘high’ EVs purity and it was therefore selected for the subsequent functional experiments (Figure S1C).

### 3.2 Cardiac Endothelial Cells Rapidly Internalise sEVs Compared to Cardiomyocytes

**FIGURE 2:**
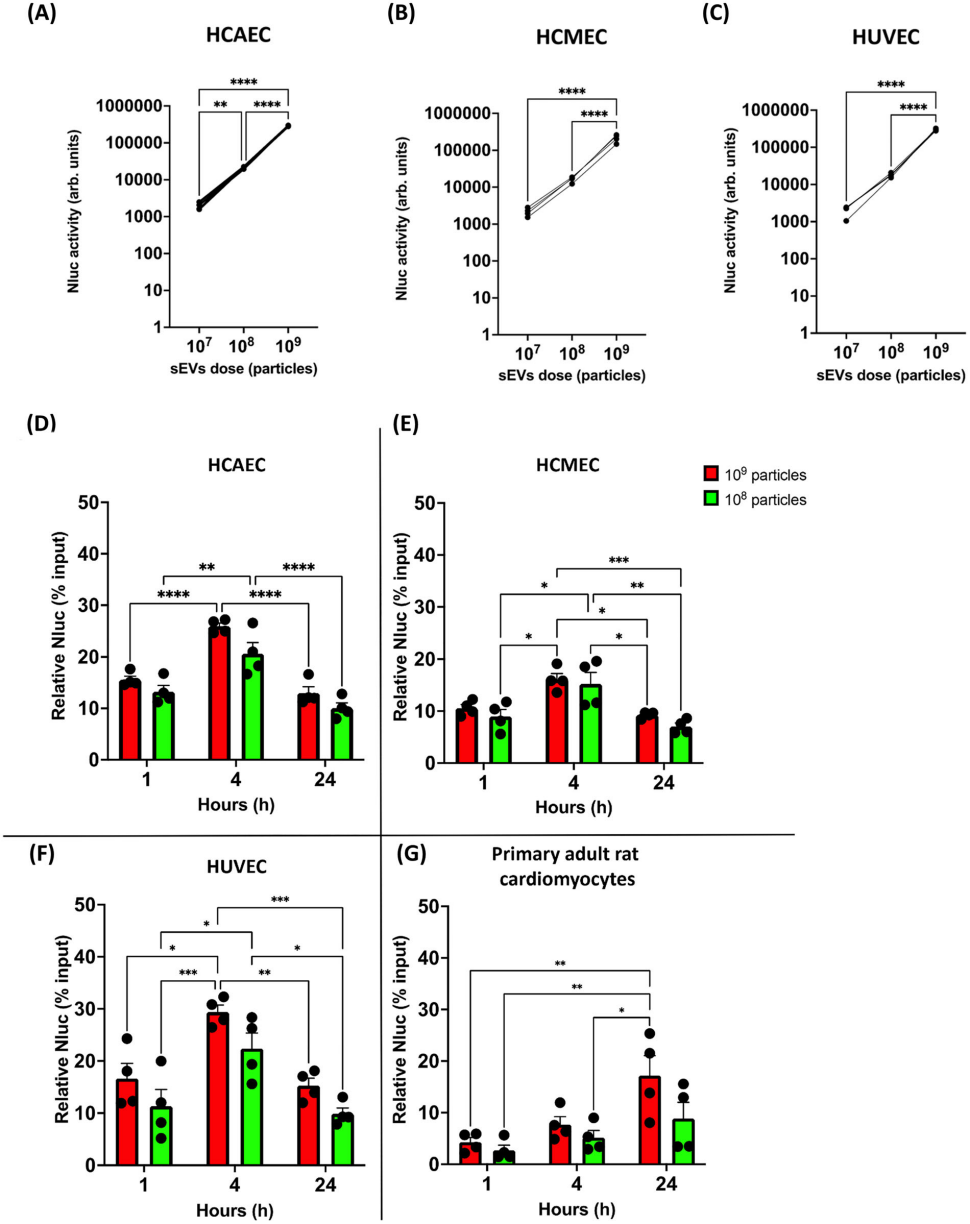
Dose‐response and time‐response uptake of HEK293‐sEVs in human endothelial cells and cardiomyocytes. (A–C) Human coronary artery and coronary microvascular endothelial cells (HCAEC and HCMEC respectively) and human umbilical vein endothelial cells (HUVEC) were treated for 4 h with increasing doses of HEK293‐sEVs. A linear dose‐response was revealed in all endothelial cells, as a 10‐fold increase of sEVs dose results in a 10‐fold increase of cellular uptake (Nluc Nanoluciferase activity). (D–F) sEVs rapidly internalise endothelial cells ((D) HCAEC, (E) HCMEC, and (F) HUVEC) within the first hour of incubation reaching the highest intracellular localisation after 4 h, corresponding to higher Nluc activity. A reduction in luciferase activity after 24 h is observed in all endothelial cells, reflective of the reduced amount of sEVs intracellularly. (G) Uptake of HEK293‐sEVs in adult rat cardiomyocytes is slow and time‐dependent, and high Nluc activity is recorded only after 24 h of sEVs incubation. Data shown as mean ± SEM (*n* = 4). *p < 0.05, **p < 0.01, ****p < 0.0001.

Next, we compared the extent to which cardiomyocytes and cardiac endothelial cells internalise sEVs. HEK293‐sEVs were incubated with primary human cardiac endothelial cells (HUVEC, HCMEC, and HCAEC) and primary adult rat cardiomyocytes for up to 24 h. After 4 h, a dose‐response curve revealed a linear relationship of particle number with luminescence in all three endothelial cells (Figure 2A–C). Since the lowest dose of 10^7^ particles had low luminescence, and was interfering with the background, it was excluded from further analysis.

Next, we investigated the time‐course of uptake of 10^8^ and 10^9^ HEK293‐sEVs in HCAEC, HCMEC, HUVEC, and adult primary rat cardiomyocytes. All three endothelial cell types rapidly internalise sEVs within the first hour of incubation and reached to the highest level after 4 h. Cytosolic levels of sEVs were reduced by 24 h of incubation, which may reflect gradual degradation after internalisation. In contrast, cardiomyocyte uptake was lower at 1 h (∼4.3% for cardiomyocytes vs ∼15% for all endothelial cells, *n* = 4, Figures 2D **–F**) and was more time‐dependent, as high levels of sEVs were achieved only after 24 h incubation. This difference in sEVs uptake in the first hour of incubation between HCMEC and cardiomyocytes was verified with confocal fluorescent imaging of cells after incubation with HEK293‐sEVs stained with CellMask Orange, to detect their uptake. However, with this method we also noticed that the pattern of fluorescence was the same as when HCMEC were directly stained by adding CellMask Orange to the cells. This may suggest that the dye can diffuse intracellularly after sEV uptake, making this method of sEVs uptake detection less accurate (Figure S2 and S3).

To ensure that the luciferase activity detected corresponds solely to internalised sEVs and does include any vesicles that had adhered non‐specifically to the cell exterior, HCMEC and rat cardiomyocytes were treated with citric acid for 5 min, before conducting the luciferase assay. After 1 h incubation with 10^9^ HEK293‐sEVs, there was no difference in sEVs uptake before or after citric acid treatment, in either HCMEC or cardiomyocytes (Figure S4).

### 3.3 The CD36 Fatty Acid Transporter Mediates sEVs Cellular Uptake

**FIGURE 3:**
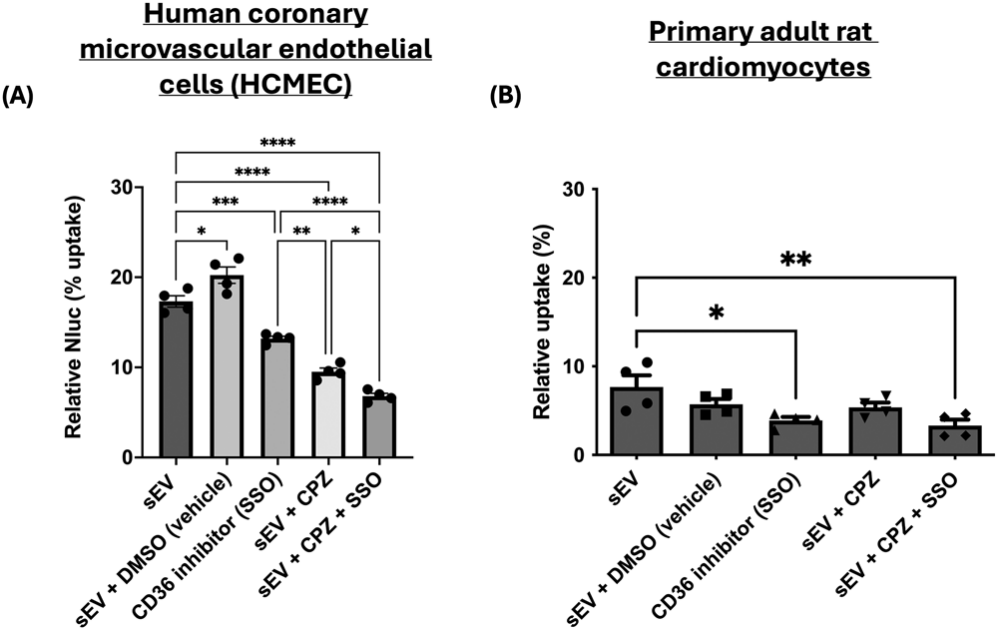
The CD36 fatty acid transporter influences HEK293‐sEVs cellular uptake. Cells were treated with 200 µM of SSO, an irreversible inhibitor for CD36, for 1 h prior to 10^9^ HEK293‐sEVs treatment for 1 h. (A) Inhibition of CD36 resulted in a reduced sEVs uptake in HCMEC. CPZ was used as an inhibitor of clathrin‐mediated endocytosis (CME), as a positive control. Combination of the two inhibitors resulted had an additive effect in the reduction of sEVs uptake. (B) Similarly in adult rat cardiomyocytes, CD36 inhibition led to a reduced Nluc activity intracellularly, indicative of a reduced HEK293‐sEVs internalisation. In this cell type, CME pathway was not involved in sEVs uptake. Data shown as mean ± SEM (*n* = 4). *p < 005, **p < 0.01, ***p < 0.001, and ****p < 0.0001. HEK293: human embryonic kidney cells 293; sEVs: small extracellular vesicles; Nluc: nanoluciferase; SSO: sulfosuccinimidyl oleate sodium; DMSO: dimethyl sulfoxide; CPZ: chlorpromazine.

We pre‐treated cells with SSO, an inhibitor of CD36, to investigate whether CD36 influences HEK293‐sEVs uptake in endothelial cells and cardiomyocytes. As a positive control, clathrin‐mediated endocytosis (CME) was inhibited using chlorpromazine. SSO significantly reduced the uptake of HEK293‐sEVs in both HCMEC and cardiomyocytes (Figure 3). More specifically, it reduced uptake in HMCEC by 25 ± 6% (*p* < 0.001, *n* = 4, Figure 3A) and in rat cardiomyocytes by 49 ± 20% (*p* < 0.03, *n* = 4, Figure 3B). CME inhibition in HCMEC reduced sEVs uptake by 45 ± 5% (*p* < 0.0001 vs. control, Figure 3A). Inhibition of both pathways simultaneously conferred additive inhibition of sEVs uptake, by 60 ± 3% vs control (*p* < 0.0001, *n* = 4), 46 ± 5% vs CD36 inhibition (*p* < 0.0001, *n* = 4) and 28 ± 9% vs CME inhibition (*p* < 0.05, *n* = 4) (Figure 3A). Interestingly, CME inhibition did not influence sEVs uptake in primary adult rat cardiomyocytes (Figure 3B).

### 3.4 The Role of CD36 in sEV Biodistribution *in Vivo*

An understanding the biodistribution and clearance of sEVs is paramount for their use in clinical practice. We therefore assessed the biodistribution of luminescence following HEK293‐sEV administration to mice. The aim was to understand: the proportion of the initial dose of sEVs that internalised into cells of the major organs examined; determine the proportion of sEVs that are eliminated by saline perfusion and were therefore located within the vascular lumen of the tissues; and assess how inhibition of CD36 influences sEV biodistribution.

**FIGURE 4:**
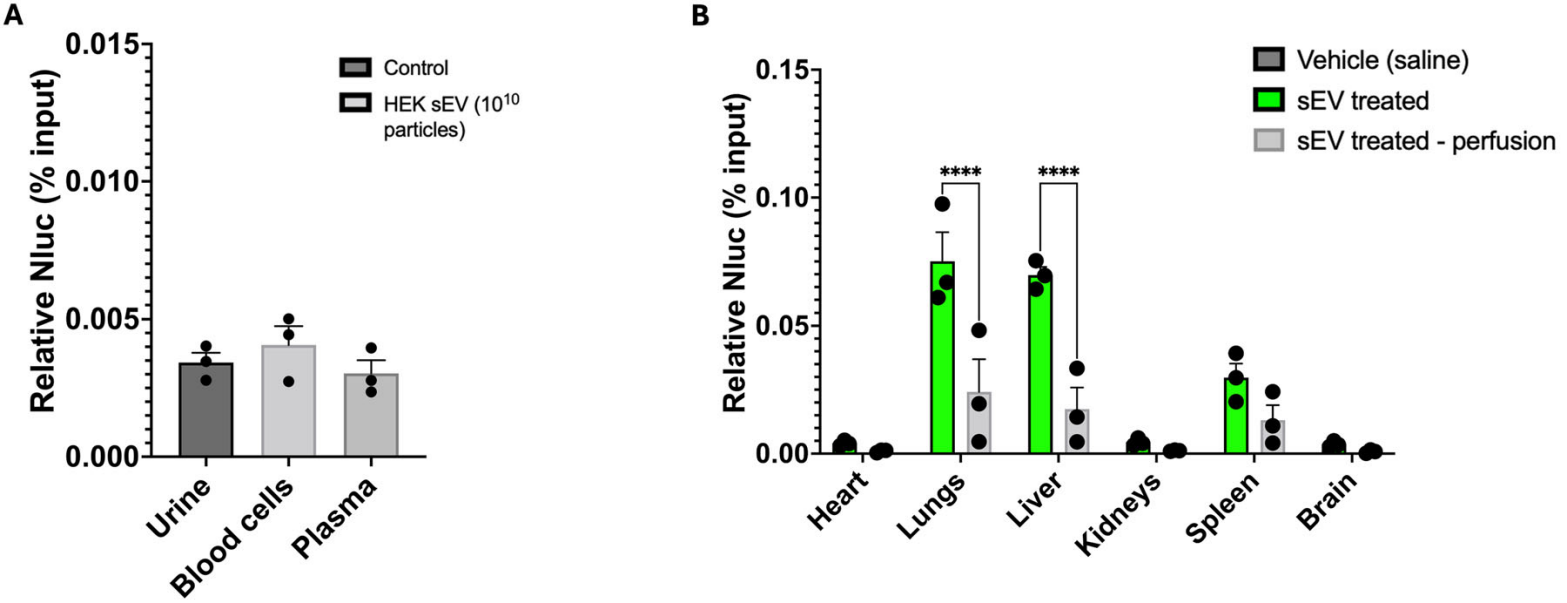
Effects of whole‐body perfusion in HEK293‐sEVs biodistribution. C57BL/6 mice were administered with 10^10^ HEK293‐sEVs and biodistribution was allowed for 1 h, prior to organ and biofluid collection and Nluc quantification in vitro. (A) Less than 0.005% of the initial administered dose was found in either urine, blood cells, or plasma of mice. (B) Major organs of HEK293‐sEVs uptake were the lungs, liver, and the spleen, where Nluc activity was less than 0.1% of the total initial dose injected. Whole‐body perfusion with saline reduced the Nluc activity recorded in these organs substantially, to levels below 0.05%. Data shown as mean ± SEM (*n* = 3). ****p < 0.0001. HEK293: human embryonic kidney cells 293; sEVs: small extracellular vesicles; Nluc: nanoluciferase.

Mice were treated with 10^10^ HEK293‐sEV intravenously, and biodistribution was allowed for 1 h, after which organs were harvested, lysed and luminescence was quantified using a plate reader. Some mice were perfused with saline before harvesting the organs to remove the blood from the vascular lumen. After 1 h, > 99% of the initial dose of sEVs was cleared from the circulation and distributed to the organs. Luminescence (reflecting HEK293‐sEV uptake) was detected primarily in the lungs, liver, and the spleen, with the highest signal in the lungs. Whole‐body saline perfusion significantly decreased luminescence in the lungs and liver (*n* = 3, *p* < 0.0001, Figure 4), suggesting that a significant proportion of the luminescence in the non‐perfused organs is in fact retained in blood compartments in the capillaries of the organs.

**FIGURE 5:**
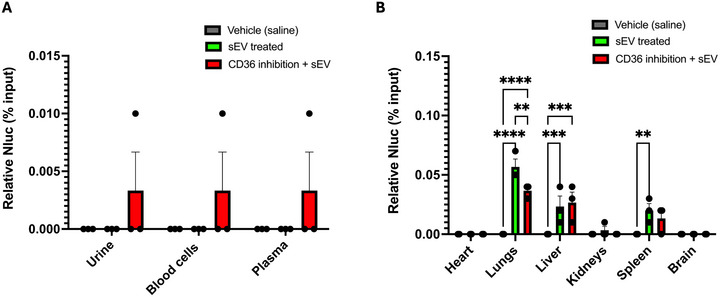
Inhibition of the CD36 transporter reduces HEK293‐sEVs biodistribution in the lungs. 12–14 weeks old C57BL/6 male mice were treated with 40 mg/kg of SSO for 1 h before the administration of 10^10^ HEK293‐sEVs. Biodistribution of sEVs was allowed for 1 h, after which organs and biofluids were collected for the in vitro quantification of the luciferase activity. (A) Nluc activity was less that 0.005% of the total Nluc activity of the initial dose injected all types of biofluids collected. (B) Major organs of sEVs distribution were the lungs, liver, and spleen, in accordance with previous experiments, and global inhibition of CD36 led to a reduction in sEVs uptake in the lungs (**p < 0.01 vs sEVs treated). Data shown as mean ± SEM (*n* = 3). ****p < 0.0001. HEK293: human embryonic kidney cells 293; sEVs: small extracellular vesicles; Nluc: nanoluciferase; SSO: sulfosuccinimidyl oleate sodium.

We next investigated how systemic CD36 blockade affects sEV distribution *in vivo*. CD36 inhibition significantly reduced sEV uptake in the lungs compared to sEV‐treated controls (*p* < 0.01, *n* = 3; Figure 5).

### 3.5 HEK293‐sEVs are Cardioprotective in a Mouse Model of Acute I/R Injury

**FIGURE 6:**
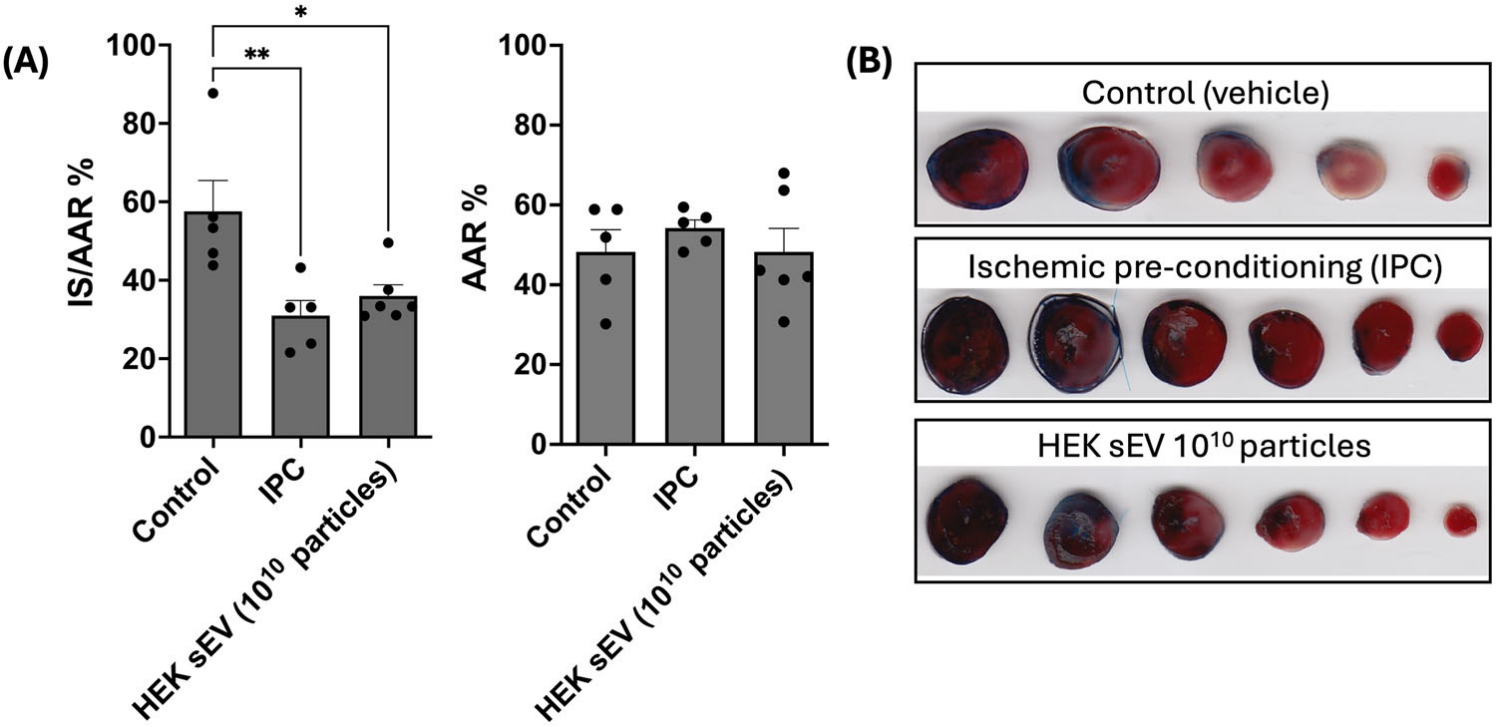
HEK293‐sEVs protect the heart in acute myocardial infarction. C57BL/6 male mice of 12–14 week old were subjected to acute myocardial ischaemia‐reperfusion injury (M I/R). Ischaemia was induced with the ligation of the left anterior descending artery of the left ventricle for 40 min, following by reperfusion for 120 min. 1010 HEK293‐sEVs were injected intravenously at the onset of reperfusion. (A) Administration of HEK293‐sEVs reduced the infarct size compared to control (36 ± 3 % vs. 58 ± 8% respectively, *p < 0.05, *n* = 5). As a positive control, an IPC (ischaemia pre‐conditioning) group was included, which reduced the infarct size to similar levels with HEK293‐sEVs (31 ± 4%, **p < 0.01 vs. control). No difference in the %AAR was observed in all groups. (B) Representative scanned images of the excised and stained hearts for each group. Following M I/R, hearts were excised and stained with Evan's blue, for the staining of the live, undamaged, tissue, and TTC (triphenyl tetrazolium) for the staining of the area at risk (AAR, red). The ischaemic (dead) tissue appears as white/pale. Data shown as mean ± SEM (*n* = 5). *p < 0.05 and **p < 0.01. HEK293: human embryonic kidney cells 293; sEVs: small extracellular vesicles; IS: ischaemic size; AAR: area at risk.

Next, we assessed the ability of HEK293‐derived sEVs to limit infarct size in a mouse model of myocardial infarction. Administration of 10^10^ HEK293‐sEVs at the onset of reperfusion in mice subjected to acute myocardial ischaemia‐reperfusion injury significantly reduced the infarct size by >20% (36% ± 3 % vs. 58% ± 8% for controls, *p *< 0.05, *n* = 5, Figure 6A,B).

### 3.6 CD36 Inhibition Abrogates Cardioprotection by HEK293‐sEVs in I/R

**FIGURE 7:**
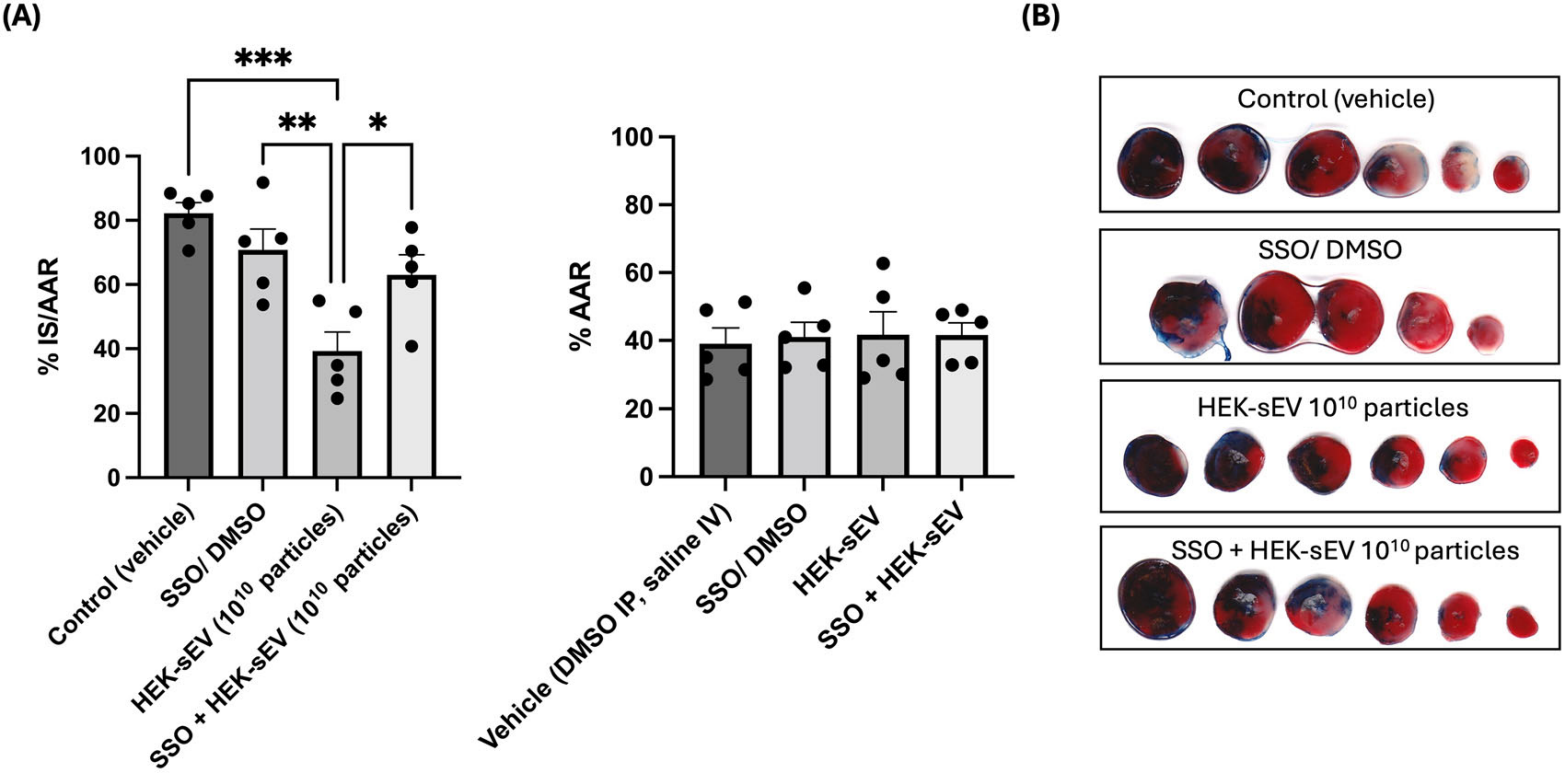
Inhibition of CD36 reverts the cardioprotective effects of HEK293‐sEVs. Mice were subjected to myocardial ischaemia‐reperfusion injury (M I/R; 40 min of ischaemia followed by 120 min of reperfusion). To observe the effects of the CD36 fatty acid transporter in HEK293‐sEVs cardioprotection, the irreversible inhibitor SSO was administered intraperitoneally at 40 mg/kg 1 h before the injection of HEK293‐sEVs (dose: 1010 particles at the onset of reperfusion). (A) sEVs reduced the infarct size compared to control (49 ± 6 % for HEK293‐sEVs vs. 82 ± 3 % for control, ***p < 0.001) and inhibition of CD36 abrogated that effect (63 ± 6 % for HEK293‐sEVs + SSO vs. 39 ± 6 % for HEK293‐sEVs, *p < 0.05). Administration of the inhibitor alone did not affect the infarct size. (B) Cross sections of the hearts for each group. Hearts were stained to visualise the alive tissue (Evan's blue), and then incubated with TTC (triphenyl tetrazolium) to stain the area at risk (red). Subsequently, the dead tissue was visualised in pale/white colour. Data shown as mean ± SEM (*n* = 5). *p < 0.05, **p < 0.01, and ***p <0.001. HEK293: human embryonic kidney cells 293; sEVs: small extracellular vesicles; IS: ischaemic size; AAR: area at risk; SSO: sulfosuccinimidyl oleate sodium.

Since we had seen a role for CD36 in uptake and biodistribution of HEK293‐sEVs, we next investigated whether it is required for cardioprotection. Global inhibition of CD36 was established by administration of SSO, prior to ischaemia and reperfusion, with vehicle or 10^10^ sEVs then administered at reperfusion as previously. SSO alone did not alter the infarct size compared to control (70% ± 7% vs. 82% ± 3% for controls, *n* = 5, Figure 7A,B). However, it completely abrogated the cardioprotective effects of HEK293‐sEVs (63% ± 6% for HEK293‐sEVs + SSO vs. 39% ± 6% for HEK293‐sEVs, *p* < 0.05, *n* = 5, Figure 7A,B).

## 4 Discussion

Our results demonstrate that we can achieve highly enriched sEVs from HEK293 cell media by using tangential flow filtration coupled with SEC, as assessed by particle/protein ratio (Webber and Clayton 2013). This is important, in order to be able to attribute the observed effects to the vesicles rather than any co‐isolated impurities. We observed a very close dose‐response relationship between the quantity of HEK293‐derived sEVs and cellular luminescence after incubation of endothelial cells with the sEVs. Notably, endothelial cells internalised sEVs more rapidly than cardiomyocytes. This is particularly notable considering that the internal volume of a single ventricular cardiomyocyte is approximately 10 ‐ 20 times greater that of an endothelial cell. As such, it can be calculated that, after addition of 10^9^ HEK293‐sEVs to cells, the total contents of sEVs taken up after 1 h (see Figure 3) will be diluted ∼20 ‐ 40 ‐ fold within the total cytosolic volume of endothelial cells in the dish (Figure S5). On the other hand, when 10^9^ sEVs HEK293‐sEVs are added to cardiomyocytes those that are taken up will be diluted ∼330‐fold within the cytosolic volume, suggesting their overall biochemical effects may be less than in endothelial cells. An additional factor to consider after *in vivo* delivery is the non‐fenestrated endothelial barrier of the heart, which obstructs sEVs from reaching the underlying cardiomyocytes.

Following *in vivo* administration, sEVs rapidly disappear from the blood, within the first few minutes of injection (Kang et al. 2021). In rat cardiomyocytes, we found that higher sEVs uptake only occurred with prolonged sEVs incubation (24 h). Since sEVs biodistribution and clearance from the circulation is very rapid, it is unlikely that cardiomyocytes internalise meaningful amounts of sEVs *in vivo*, in comparison to cardiac endothelial cells. Generally, it is still ambiguous if the therapeutic effects of sEVs are mediated by receptor interactions on the cell membrane, or by the delivery of their cargo intracellularly, and how much of that cargo is needed to exert a biological effect. If these effects are dependent on cargo delivery, then the increased sEVs internalisation in endothelial cells at shorter time‐periods may suggest that the endothelium could be an important mediator of the sEVs‐mediated therapeutic potential. Additionally, it has been reported that removal of the endothelium in an *ex vivo* model of isolated hearts, abrogated the cardioprotective effects of sEVs (Penna et al.), enhancing the argument that indeed the endothelial cells may be a key mediator of sEVs‐mediated cardioprotection. However, the exact mechanisms by which the endothelium exerts this sEVs‐mediated cardioprotection remains to be elucidated.

Interestingly, in our biodistribution experiments, we found that whole‐body perfusion reduced bioluminescence in the major organs of localisation (lungs, liver, spleen). This suggests that in reality, a significant quantity of sEVs remain in the blood within the organs’ vasculature rather than being taken up by the cells. This should be taken into account in biodistribution studies of sEVs.

In line with reports on other types of sEV (Kang et al. 2021, Gupta et al. 2020, Lázaro‐Ibáñez et al. 2021), we found that HEK293‐sEVs are rapidly distributed to the lungs, liver, and the spleen in mice after intravenous injection. This can potentially be explained by the uptake of sEV from the resident macrophages of the alveolar macrophages in the lung or the phagocytic Kupfer cells in the liver (Lai et al. 2014). Additionally, both the liver and the spleen are characterised by a discontinuous endothelium, which may facilitate the transportation of small particles, like sEV, into the cells of the tissue (Gupta et al. 2023). Even though HEK293‐sEVs are able to be taken up by cardiac endothelial cells *in vitro*, we were unable to detect significant Nanoluciferase activity in the hearts *in vivo*, even after administration of 10^10^ sEVs. Since cardiac luminescence was close to the limit of detection, the effect of CD36 inhibition on cardiac uptake could not be reliably determined. Notably, the nanoluciferase activity recorded in the organs was lower than the initial injected dose (<0.1% of the total Nluc activity of initial dose of 10^10^ HEK293‐sEVs), which could be attributed to the rapid clearance of sEVs by monocytes in the circulation, and by the intracellular lysosomal activity in the tissues. One limitation of the in vivo biodistribution experiments is that we did not investigate doses higher than 10^10^ HEK293‐sEVs, which could have resulted in elevated nanoluciferase activity in all organs, including the heart. However, this dose of sEVs was selected in order to align with the dose used in the in vivo myocardial I/R experiments. More timepoints would have been beneficial to show how the biodistribution profile of sEVs may change in the short‐term (for example between the point of injection and up to the first hour).

A limitation is that we did not investigate the how myocardial I/R alters the biodistribution profile of HEK293‐sEVs. However, in a mouse model of myocardial I/R (45 min ischaemia followed by 1 h of reperfusion) it was shown that uptake of CDC‐derived sEVs is increased in the heart, liver, and the brain after I/R in mice (1 h biodistribution; sEVs injected i.v. at reperfusion) (Ciullo et al. 2022).

With studies of sEV uptake and distribution it is important to consider effects of the label used. Transfection with the CD63‐Nluc plasmid has been previously used for similar studies, but this approach does increase expression of CD63 on the sEV membrane, which can potentially influence their biodistribution (Lázaro‐Ibáñez et al. 2021). Although we did not compare the biodistribution of modified versus unmodified HEK293‐sEVs, we showed in a previous study that CD63 overexpression did not alter the size of the vesicles (Sulaiman et al. 2025). Notably it was previously shown that sEV overexpressing CD63 are distributed more in the lungs compared to the rest of the sEV subpopulation (Lázaro‐Ibáñez et al. 2021). A commonly used alternative is to use lipophilic dyes (e.g., PKH7, or DiR) to track sEVs, but we and others have demonstrated that these are liable to artefacts, such as fluorescent micelles and aggregates of a size similar to EV, and they thus provide unreliable results for uptake and biodistribution experiments (Takov et al. 2017, Simonsen 2019). We therefore believe that Nanoluciferase tracing is a superior method, although it only tracks the sEV that carry the CD63‐Nluc construct. Also, Nanoluciferase reports enzyme presence which could correspond to either intact or degraded sEV intracellularly, or the free CD63‐Nluc construct released by the sEV. Notably, it has previously been shown that the free, recombinant Nluc is unable to enter the cells (Bonsergent et al. 2021), supporting our argument that the detected Nluc signal corresponds to sEVs bearing the CD63‐Nluc construct. Another approach that has been used is to trace unique miRNA/siRNA content of sEVs (Ciullo et al. 2022).

Generally, for cardioprotection studies, sEVs are derived from cellular sources such as MSC and iPSC, which are known to have immunomodulatory and regenerative properties that are partially ascribed their secreted vesicles. HEK293 cells, on the other hand, lack these properties. However, when HEK293‐sEVs were administered to mice, they reduced the infarct size in a model of acute ischaemia‐reperfusion injury. This suggests that sEVs from apparently biologically ‘inert’ cell sources may yet hold promising therapeutic potential. The sEVs were administered immediately prior to reperfusion, and the majority of the cell death resulting in infarction occurs rapidly within the first ∼15 min of reperfusion, therefore it is highly unlikely that that there is sufficient time for miRNA cargo to have an effect. We propose that protein or lipid content of HEK293‐sEVs may mediate protection, though exact mechanism by which HEK293‐sEVs protect the heart has not been established. One hypothesis is that these cardioprotective effects may be mediated by phosphatidylserine (PS), a lipid component of the sEV membrane (Llorente et al.). This is based on the fact that PS‐presenting synthetic liposomes mediate cardioprotection in a mouse model of permanent LAD occlusion (Harel‐Adar et al. 2011) and suggestions in the existing literature that CD36 mediates uptake of PS‐presenting vesicles (Willekens et al. 2005, Matsumoto et al. 2017).

Most studies to date that have examined whether sEVs can reduce infarct size acutely (i.e.: when assessed up to 48 h after reperfusion) have used EVs obtained from MSCs that were grown *in vitro* (Davidson and Yellon 2018, Takov et al. 2020). Cardiosphere‐derived cells have also been shown to reduce infarct size in pigs subject to IR (Gallet et al. 2017). sEV isolated from plasma are cardioprotective, but this ability is impaired by diabetes (Davidson et al. 2017, Vicencio et al. 2015). Endothelial cells also release cardioprotective exosomes, but only when healthy and not when inflamed or activated (Davidson et al. 2018, Herrera‐Zelada et al. 2023). Additionally, sEVs obtained from some, but not all neuronal stem cells are cardioprotective (Katsur et al. 2021). Interestingly, one comparison of sEV from several different cell sources, determined that embryonic stem cells were superior source of sEVs with pro‐angiogenic and anti‐fibrotic effects that might be useful for cardiac repair (González‐King et al. 2024). Although the acute cardioprotection assay is different and relies more on preventing cell death, it could indicate that sEVs are able to confer cardioprotection when obtained from embryonic cell sources, and might explain why HEK293‐sEVs are protective.

The significant similarities between sEVs with oxLDL particles, and the fact that phosphatidylserine and phosphatidylcholine are basic constituents of the vesicular membrane (Llorente et al.), led us to investigate how CD36 may influence HEK293‐sEVs function **in vitro** and **in vivo**. The CD36 fatty acid transporter, which is located on endothelial cells, macrophages, and cardiomyocytes, and mediates the uptake of oxLDL and fatty acids such as phosphatidylserine and phosphatidylcholine. It plays a crucial role in atherosclerosis, as it contributes to the excessive uptake of oxLDL from macrophages, mediates inflammation and the formation of foam cells (Ackers et al. 2018, Li et al. 2020, Sato et al. 2018, Yang et al. 2018). Additionally, CD36 on macrophages contributes to the phagocytotic clearance of dying cells thus mediating tissue repair and exerting anti‐inflammatory effects. We found that inhibition of the transporter in vitro led to a reduced sEVs uptake in both endothelial cells (HCMEC) and cardiomyocytes. For comparison, we also inhibited the CME pathway of uptake, and showed that in endothelial cells dual inhibition (CME and CD36) led to an additive reduction of HEK293‐sEVs internalisation. This finding suggests that CD36‐mediated uptake may be independent of CME. Our data from in vivo experiments showed that global inhibition of the CD36 transporter altered the biodistribution profile of sEVs (reduced uptake in the lungs) and abrogated the cardioprotective effects of HEK293‐sEVs in acute MI. Notably, in our reperfused mouse model of MI, inhibition of CD36 by itself, did not alter the infarct size compared to the vehicle group. Several other studies have previously investigated the role of CD36 itself in MI, although the data are conflicting. In the reperfused animal model of MI, a 14‐chronic or acute inhibition of CD36, led to a reduction in infarct size (Bessi et al. 2012). In contrast, in a spontaneously hypertensive rat model the overexpression of the fatty acid transporter reduced the infarct size (Neckář et al. 2012). Overall, these data highlight the pleiotropic effect of CD36 in cardiovascular pathology. Notably, in this study, it is unknown to what extent pharmacological inhibition of CD36 may have altered the action of macrophages in cardiac tissue repair. Other studies have shown an increase in myocardial damage following CD36 inhibition, but only in a permanent coronary ligation in vivo model (Lindsey et al. 2019; Dehn and Thorp 2018; DeLeon‐Pennell et al. 2016). This is different to the reperfused I/R model, where others have shown that chronic inhibition of CD36 reduced the infarct size (Bessi et al. 2012, Huynh et al. 2018, Zheng et al. 2017, Zhang et al. 2022), but our experiments showed that acute inhibition of CD36 did not alter the infarct size compared to saline control. It could be hypothesised that a chronic CD36 inhibition is more likely to influence the pleiotropic biological function of the receptor in the cardiac tissue than the short‐term.

In conclusion, we believe that our study will shed more light into crucial aspects of pharmacokinetics and pharmacodynamics of EVs, that can potentially facilitate their clinical use.

## Author Contributions

**Sean M. Davidson**: conceptualization, funding acquisition, writing – review and editing, project administration, supervision. **Elias Sulaiman**: investigation, writing and original draft, methodology, writing – review and editing. **Derek M. Yellon**: review & editing, supervision.

## Conflicts of Interest

The authors report no conflict of interest.

## Supporting information




**Supplementary Figure S1**: Physical and biochemical characterisation of HEK293‐sEV.
**Supplementary Figure S2**: Confocal microscopy of HEK293‐sEV uptake in HCMEC.
**Supplementary Figure S3**: Confocal microscopy of HEK293‐sEV uptake in primary adult rat cardiomyocytes.
**Supplementary Figure S4**: Citric acid treatment did not affect Nluc activity measurement.

## Data Availability

The data presented in this study is original and available upon request to the corresponding author.
