# Supplementary material for: The Fatty Acid Transporter CD36 Mediates Uptake, Biodistribution, and Cardioprotection by Small Extracellular Vesicles From HEK293 Cells
Source: J Extracell Vesicles. 2026 Mar 17;15(3):e70254. doi: 10.1002/jev2.70254 (PMC13097353; doi:10.1002/jev2.70254)
Supplement: Supplementary file 1 — Supplementary Figure S1: Physical and biochemical characterisation of HEK293‐sEV. Supplementary Figure S2: Confocal microscopy of HEK293‐sEV uptake in HCMEC. Supplementary Figure S3: Confocal microscopy of HEK293‐sEV uptake in primary adult rat cardiomyocytes. Supplementary Figure S4: Citric acid treatment did not affect Nluc activity measurement. [file JEV2-15-e70254-s001.docx]

Supplementary material

**The fatty acid transporter CD36 mediates uptake, biodistribution, and cardioprotection by human embryonic kidney 293 (HEK293)-derived sEV**

Elias Sulaiman, Derek M Yellon, Sean M Davidson*

The Hatter Cardiovascular Institute, University College London, 67 Chenies Mews, London WC1E 6HX, United Kingdom.

***Corresponding author.**

Professor Sean Davidson

The Hatter Cardiovascular Institute

University College London

67 Chenies Mews

London WC1E 6HX

Tel: +44 203 447 9894

email: [s.davidson@ucl.ac.uk](mailto:s.davidson@ucl.ac.uk)


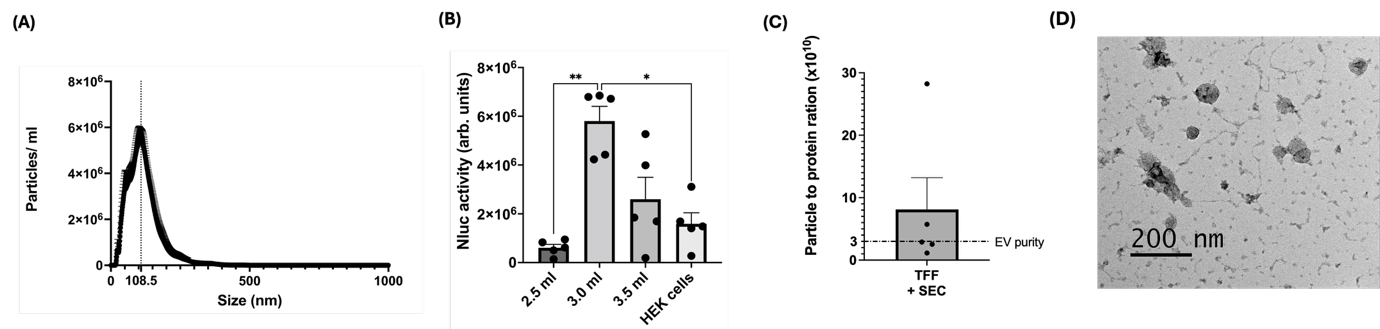


**Supplementary figure S1: Physical and biochemical characterisation of HEK293-sEV**. **(A)** Size distribution of HEK293-sEV from nanoparticle tracking analysis (NTA), reveal a narrow particle distribution less than 200 nm. **(B)** Luciferase assay of SEC fractions and HEK293 cells reveal that the most particle-rich fraction (3.0 ml) is the most luminescent of (high Nluc activity). **(C)** The purity of HEK293-sEV isolations with TFF and SEC is high, according to Webber calculations. **(D)** Transmission electron microscopy image (TEM) of HEK293-sEV showing small cup-shaped vesicles. Scale 200 nm.

**Supplementary figure S2: Confocal microscopy of HEK293-sEV uptake in HCMEC**. **(A)** HEK293-sEV were stained with Cellmask Orange plasma membrane dye and incubated in HCMEC for 1 hour. **(B)** As a control group, the pellet of the dye only was incubated in HCMEC for 1 hour, to exclude the possibility that the molecules of the dye do not form micelles. **(C)** HCMEC were directly stained with the dye for 5 minutes or **(D)** for 1 hour, to show that in prolonged periods the dye diffuses from the membrane and the staining has a similar pattern with (A).

**Supplementary figure S3:** **Confocal microscopy of HEK293-sEV uptake in primary adult rat cardiomyocytes**. **(A)** HEK293-sEV were stained with Cellmask Orange plasma membrane dye and incubated in cardiomyocytes for 1 hour. **(B)** As a control group, the pellet of the dye only was incubated in cardiomyocytes for 1 hour, to exclude the possibility that the molecules of the dye do not form micelles. **(C)** Rat cardiomyocytes were directly stained with the dye for 5 minutes or **(D)** for 1 hour, to investigate how the dye diffuses from the cell membrane to other lipid organelles after prolonged periods.

**Supplementary Figure S4: Citric acid treatment did not affect Nluc activity measurement**. HCMEC and primary adult rat cardiomyocytes were incubated with 10^9^ particles for either 1 hour (HCMEC) or at three different timepoints (rat cardiomyocytes). **(A)** Citric acid treatment after 1 hour incubation of HEK293-sEV did not change the quantified Nluc activity and in HCMEC. **(B)** Similarly, citric acid treatment resulted in similar Nluc activity compared to control for all three timepoints in rat cardiomyocytes.
